# Tumor mimicking cervicocranial actinomycosis with intracranial extension- a diagnosic dillema-first case report in somalia

**DOI:** 10.1016/j.radcr.2024.10.086

**Published:** 2024-11-16

**Authors:** Abdikadir Mohamed Dirie, Ismail Gedi Ibrahim, Shuayb Moallim Ali Jama, Faisal Abdi Osoble Osman, Yahye Garad Mohamed, Abdinasir Mohamed Elmi, Ahmed Adam Osman, Mohamed Osman Dahir, Abdirahman Mohamud Haji Ali, Ismail Mohamoud Abullahi, Nuh Mohamed Yahye, Mohamed Salad Kadiye

**Affiliations:** a Radiology Department, Mogadishu Somali Turkey, Recep Tayyip Erdogan Training and Research Hospital, Mogadishu, Somalia; b Pathology Department, Mogadishu Somali Turkey, Recep Tayyip Erdogan Training and Research Hospital, Mogadishu, Somalia; c Ophthalmology Department, Mogadishu Somali Turkey, Recep Tayyip Erdogan Training and Research Hospital, Mogadishu, Somalia

**Keywords:** Actinomycosis, Cervicofacial, Malignancy, Thoracopulmonary, CT tomography, Magnetic resonance imaging (MRI)

## Abstract

Actinomycosis is a rare bacterial infection characterized by nonspecific clinical and radiological manifestations. It often presents in cervicofacial, abdominopelvic, and thoracopulmonary forms, with cervicofacial being the most common. The extensive differential diagnosis and vague clinical and radiological features contribute to diagnostic challenges. Cancer, tuberculosis (TB), and nocardiosis are commonly considered in differential diagnoses. This report presents a biopsy-confirmed case of cervicocranial actinomycosis with intracranial extension.

## Introduction

Actinomycosis, a rare infection caused by Gram-positive filamentous anaerobic or facultative anaerobic bacteria from the *Actinomycetaceae* family, primarily inhabits the oropharynx, gastrointestinal tract, and female genital tract [1]. Cervicofacial presentations account for approximately 55% of actinomycosis cases [2,3].

CNS involvement typically occurs through metastatic hematogenous spread or direct extension from the base of the skull, though bone involvement is rare [3,4]. Risk factors include dental issues, head trauma, sinusitis, chronic mastoiditis, ventricular septal defects, immunosuppression, and infections related to intrauterine devices [3,1]. Diagnosis is challenging due to the nonspecific clinical and radiological features of actinomycosis [5], and other etiologies such as cancer, TB, and nocardiosis are frequently considered. CT imaging often demonstrates soft tissue lesions and bone involvement [6], while MRI provides more detailed information on parenchymal and meningeal disease. However, MRI findings in intracranial actinomycosis are underreported [4]. Definitive diagnosis relies on histopathological confirmation [7]. Treatment typically involves surgical intervention and long-term antibiotic therapy, which yields positive outcomes [8].

This case report presents an extremely rare instance of cervicocranial actinomycosis, initially misdiagnosed as malignancy, with diagnosis confirmed through histopathological examination.

## Case report

A 26-year-old male presented to the emergency department with complaints of left occipital and posterior cervical pain and swelling, reportedly following a military training injury sustained 6 months prior. The patient also reported decreased hearing and a sensation of fullness in both ears.

On physical examination, ulcerations with scaling were observed in the left occipital region, along with diffuse swelling extending to the posterior cervical area. The patient had no prior medical history or comorbidities. Blood tests revealed mild CRP elevation, which decreased on subsequent visits.

**Fig. 1:**
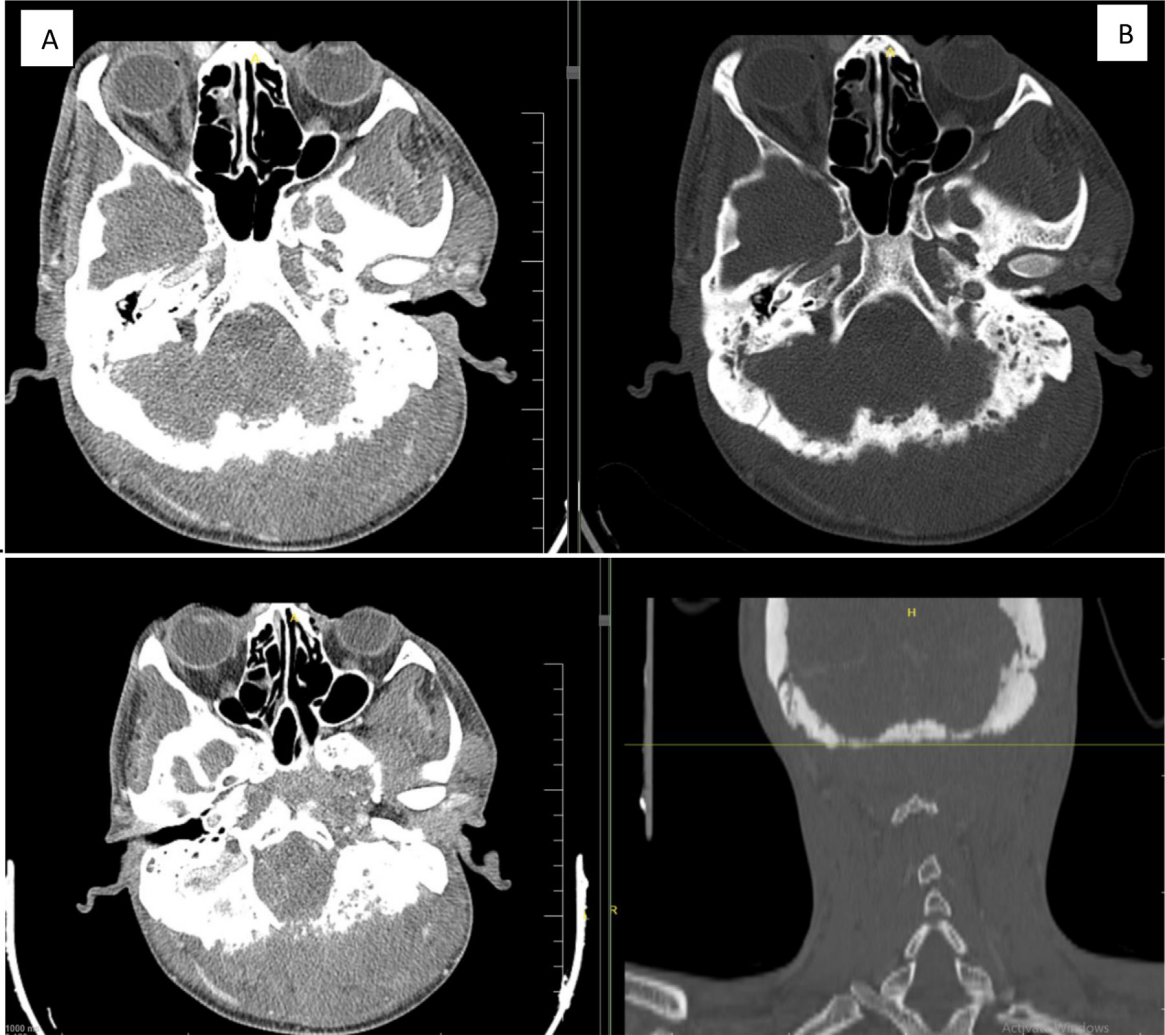
(A and C) show cerico-occipital soft tissue mass. (B and D) show disruption and sponging of the calvarium at the occipital and lefgt temporopariel bones.

**Fig. 2:**
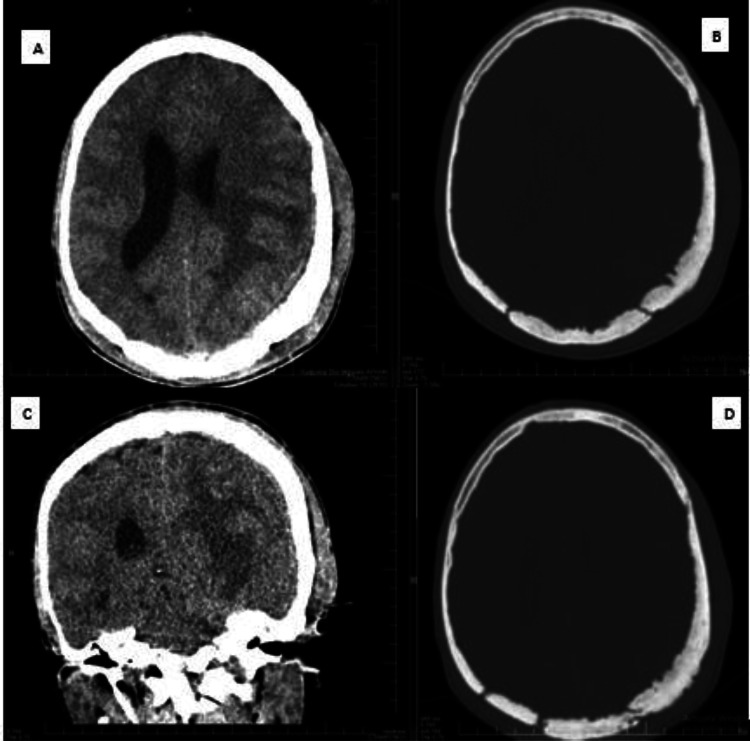
(A and C) Vasogenic edema of the left temporoparietal regions infering intracranial extension of the disease process. (B and D) osseous involvement with sponging and irregular inner and outer tablets.

**Fig. 3:**
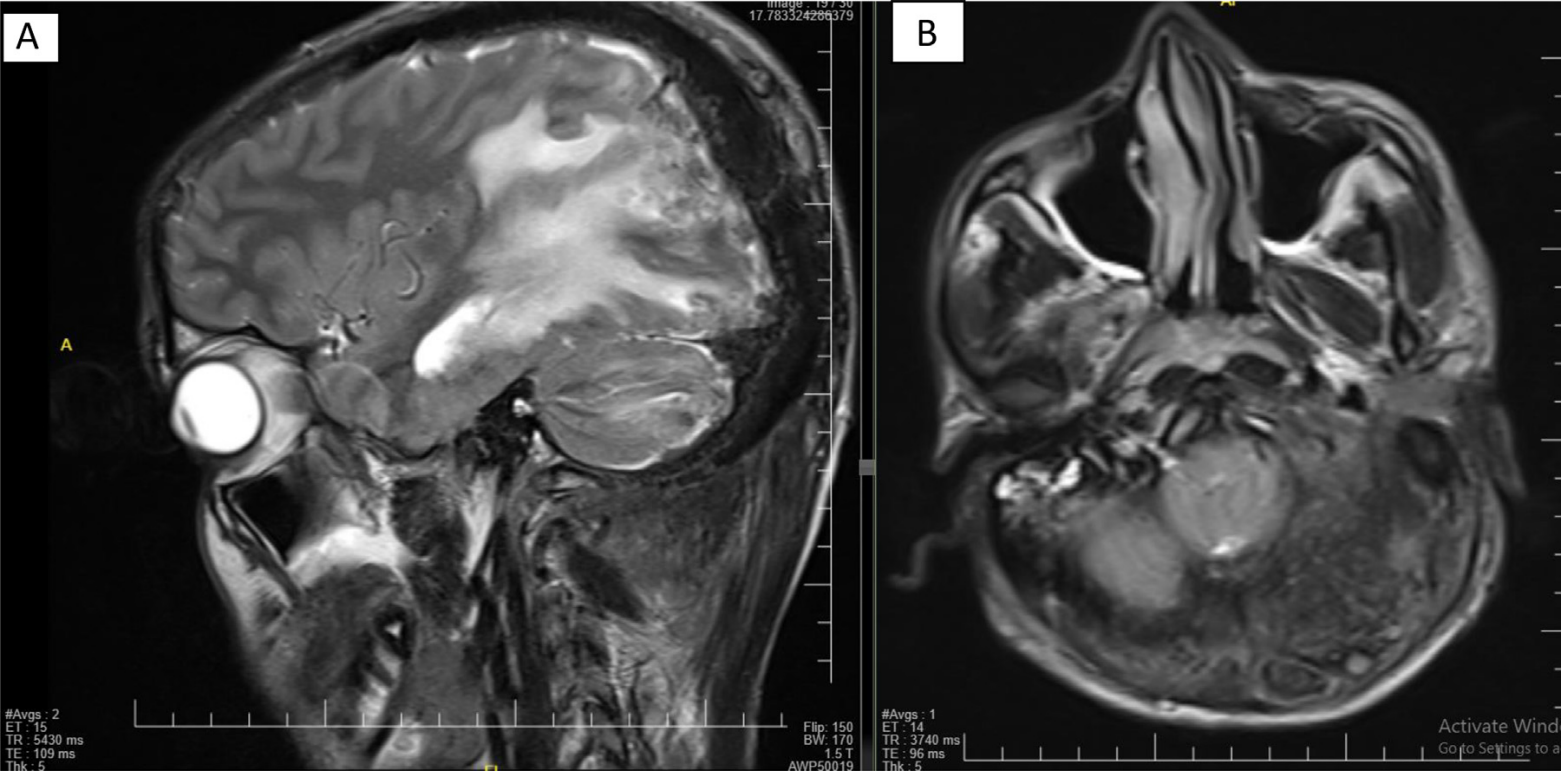
(A) Parietal occipital and left cerebellar hemisphere edema. (B) Soft tissue mass with heterogeneous signals.

**Fig. 4:**
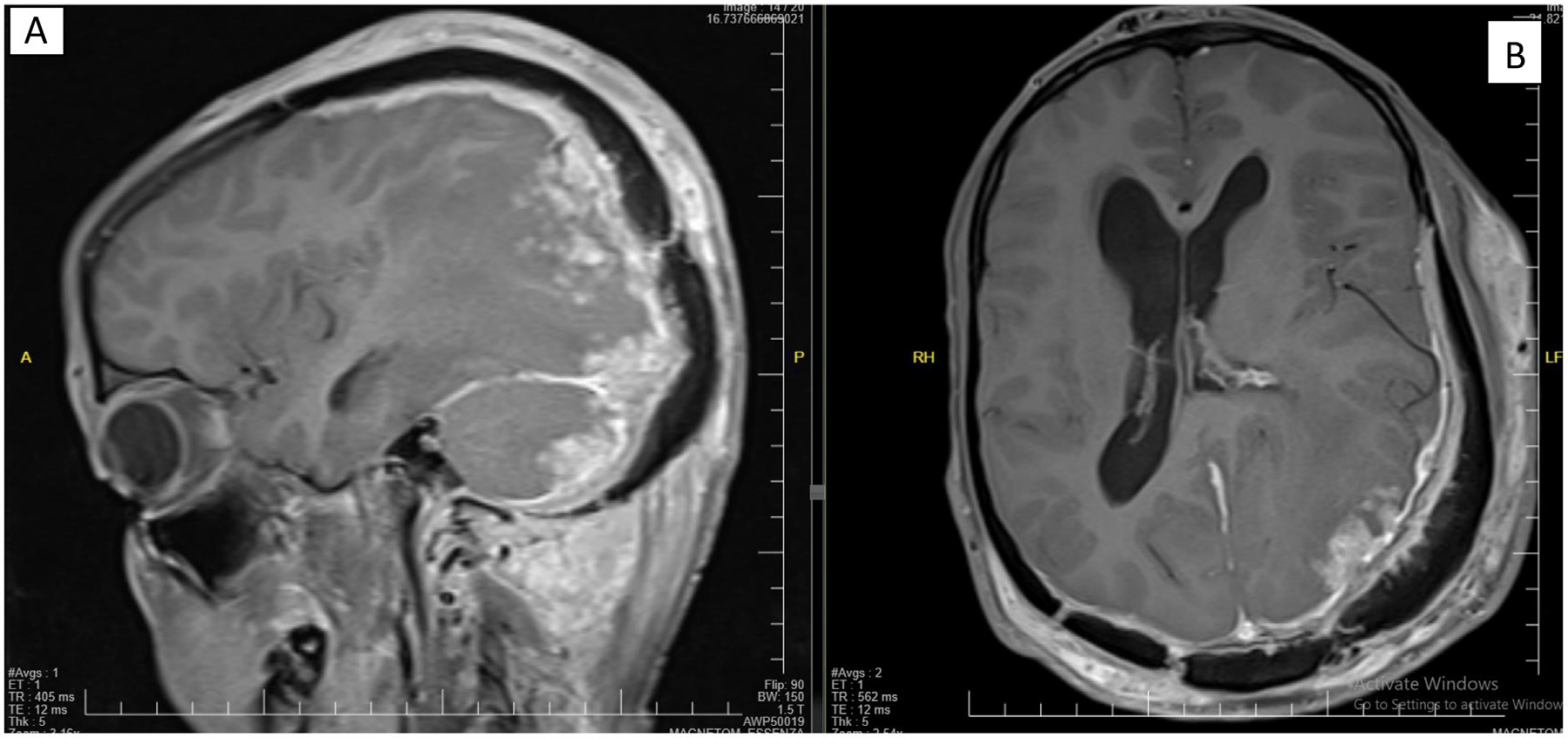
Bilateral occipital and right temporoparietal soft tissue masses with T1+C postcontrast enhancement. There is considerable intracranial extension with parenchymal and meningeal involvement.

**Fig. 5:**
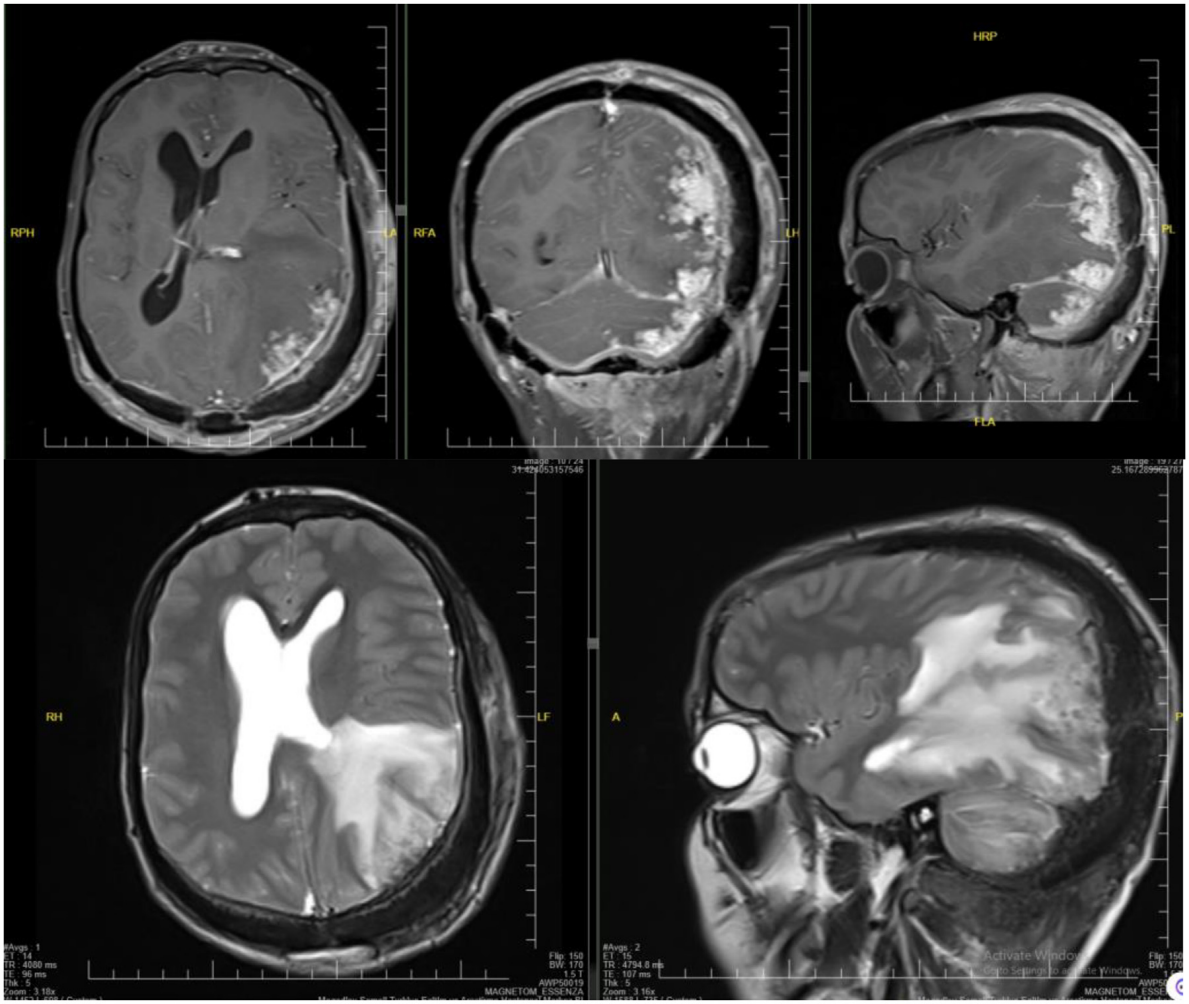
T2WI vasogenic edema with T1+C pachymeningeal thickening and enhancement with intra parenchymal serpantine enhancement. Bilateral occipital and right temporoparietal soft tissue masses with postcontrast enhancement.

**Fig. 6:**
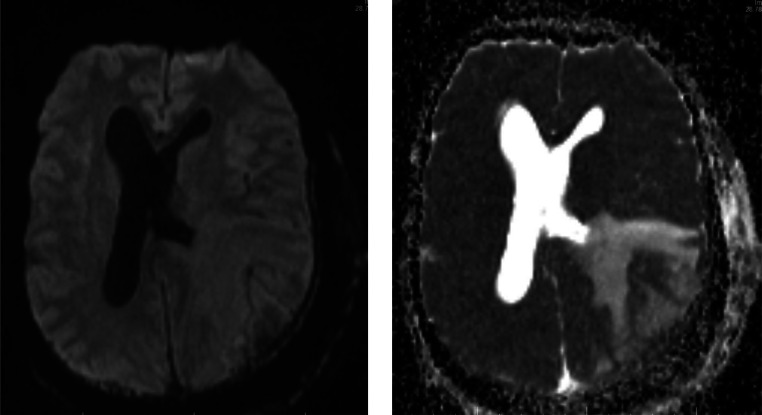
A&B DWI and ADC map show no diffusion restrictions areas.

**Fig. 7:**
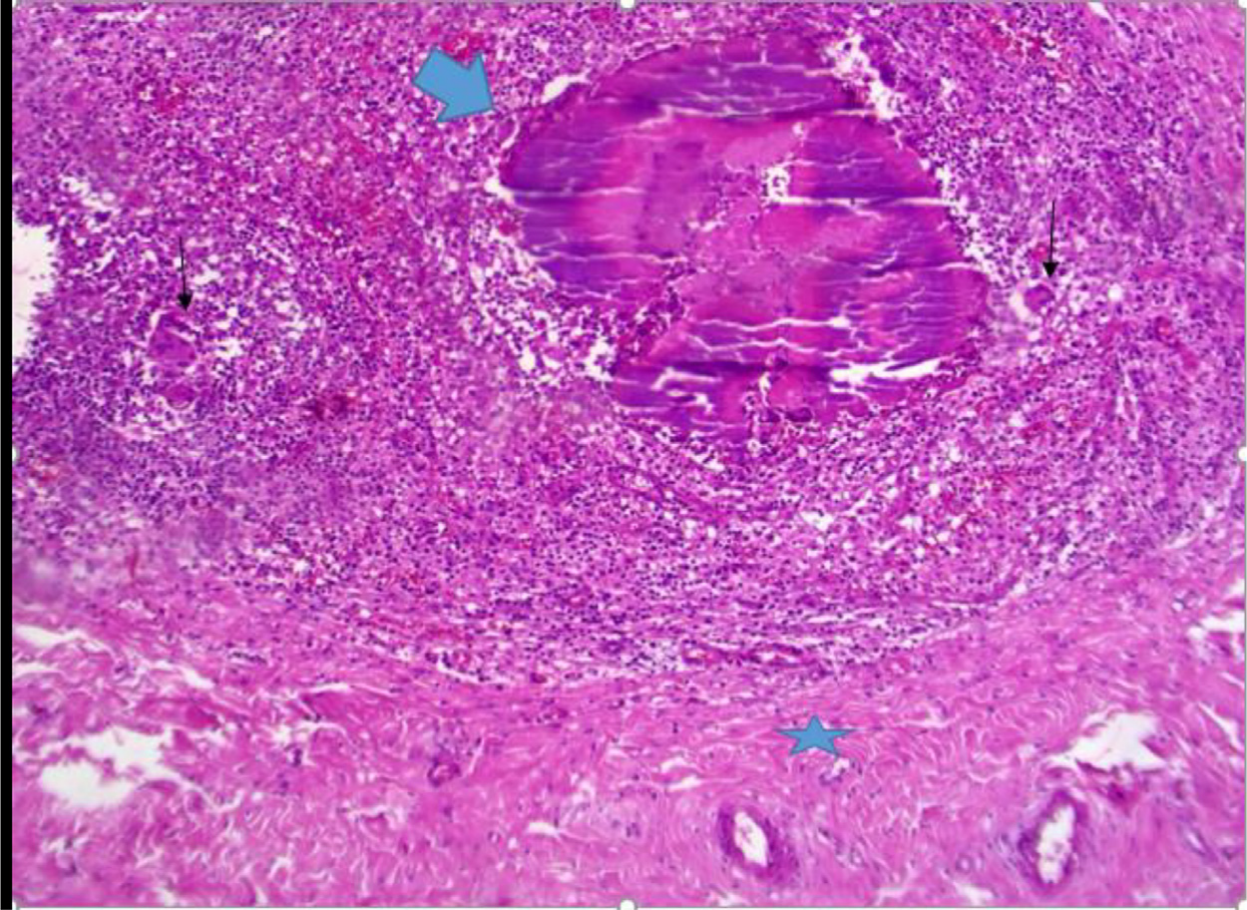
(Scalp biopsy) fibroblasts, inflammatory cells, giant cells (thin black arrow), foamy histiocytes surrounding the Bacterial colonies Sulphur granules (thick arrow). The surrounding fibrous tissue, scalp superficial fascia (star). Contributed by Dr IM Abdullahi, MD.

CT scans revealed ill-defined soft tissue masses extending from the posterior cervical to the occipital regions. Bilateral mastoid and occipital bone sponging with lytic lesions were observed. The left mastoid air cells and middle ear cavities are opacified (Fig. 1, Fig. 2, Fig. 3, Fig. 4, Fig. 5, Fig. 6, Fig. 7).

**Fig. 8:**
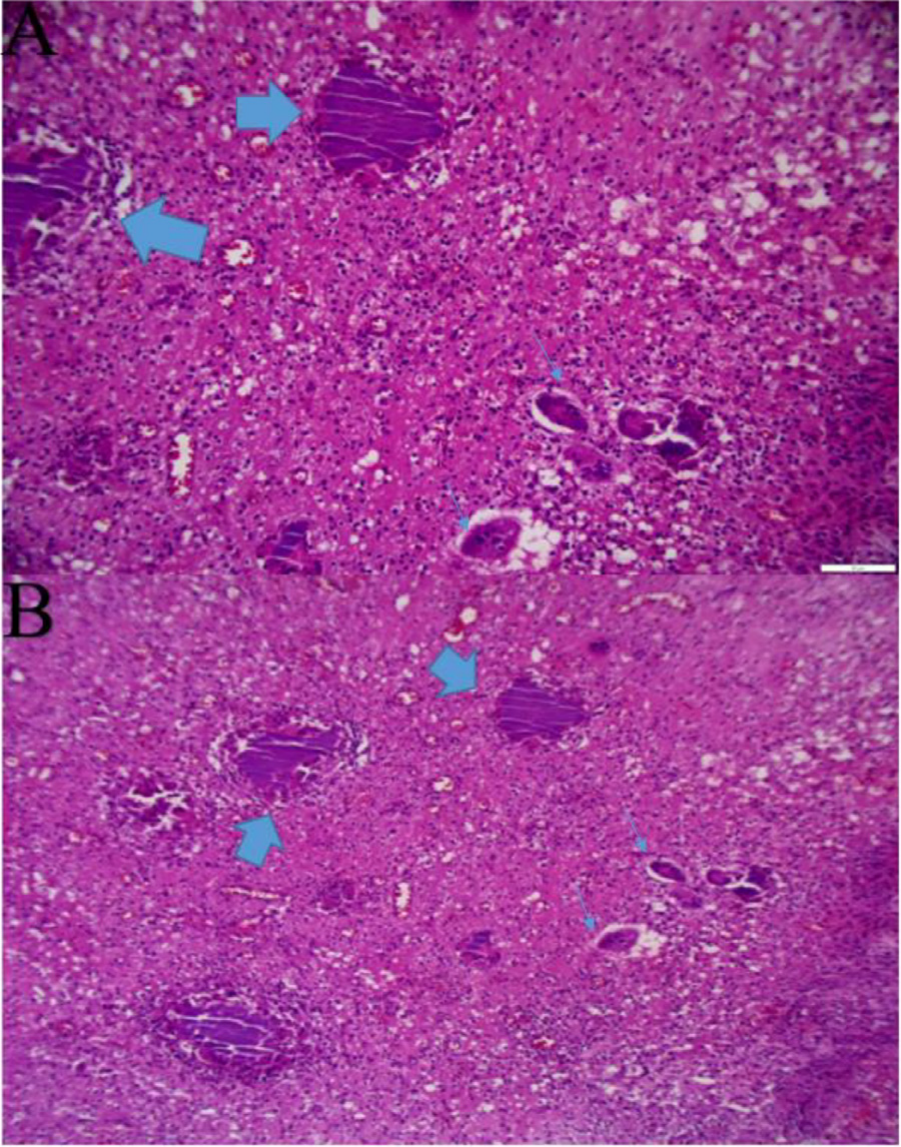
Histopathologic view; (A, B) (Brain tissue) The section shows Mixed inflammatory cells, giant cells (thin arrows), and sulfur granules (thick arrows) at the center of the inflammatory reaction, composed of basophilic radiating filaments. Intermediate and high magnification (20×, 40×). Contributed by Dr IM Abdullahi, MD.

Cervical and brain MRI scans demonstrate soft tissue thickening and edema more pronounced at the posterior cervical and left occipitoparietal regions. Postcontrast MRI showed extensive cervicocranial soft tissue lesions with intracranial extension and associated edema. Mild midline shifting to the right and compression of the left lateral ventricle due to edema of the left occipitoparietal regions are seen. No diffusion restriction is determined. Initially, a malignant soft tissue process was suspected, leading to a recommendation for histopathological confirmation. A scalp biopsy revealed mixed inflammatory cells (lymphocytes, neutrophils, and eosinophils), giant cells, fibroblasts, and bacterial colonies (sulfur granules) with basophilic radiating filaments, consistent with invasive scalp actinomycosis with bone destruction and meningeal involvement (Fig. 8).

Aggressive intravenous penicillin G treatment is started and is altered to oral penicillin, and he was advised to come regular visit after 1 month of the treatment. Unfortunately, the patient did not come for the follow-up visits and we failed to reach him in every effort we made. Therefore, post treatment imaging and clinical outcomes are not available.

## Discussion

Actinomycosis has been recognized for over a century as a rare bacterial infection caused by nonspore-forming anaerobic or microaerophilic Gram-positive bacteria of the *Actinomyces* genus [6]. The species most frequently associated with actinomycosis is *Actinomyces israelii* [9]. There are 3 primary clinical forms: thoracopulmonary, abdominopelvic, and cervicofacial, with the latter being the most common and accounts 50% of the cases [5].

Cervicofacial actinomycosis typically affects the mandible, upper maxilla, and temporomandibular joint. Intracranial involvement, particularly involving the base of the skull or temporal bone, is rare [2]. The infection is frequently misdiagnosed as neoplastic processes, including osteomyelitis associated with TB, nocardiosis, granulomas, or meningiomas [7]. Due to its low prevalence and the lack of awareness among medical professionals, diagnosis is often delayed.

Actinomycosis, often termed the “head and neck masquerader,” presents nonspecific clinical features that are frequently mistaken for malignancy [6]. Imaging findings are also nonspecific, with possible presentations including pachymeningitis, mass lesions with or without parenchymal and bone involvement, or ring-enhancing lesions [3]. In this case, pachymeningeal and parenchymal involvement were observed on MRI.

The patient's diagnosis was delayed due to an initial suspicion of malignancy, underscoring the importance of histopathological analysis for definitive diagnosis. Current treatment recommendations include prolonged antibiotic therapy and, when necessary, surgical intervention [10].

In conclusion, actinomycosis presents with nonspecific clinical and radiological features, often mimicking malignant processes. It should be considered in the differential diagnosis of such cases to prevent treatment delays and disease progression [11].

## Patient consent

We invited the patient and explained that his identifying factors such as the name, age, and other similar factors would not appear on this study. And we also clarified that there is no any financial or other beneficial purpose behind this study. Then, the patient accepted to participate in this paper and an informed participation and publication consents were obtained.

## Authors' contributions

**Abdikadir Mohamed Dirie:** Conceptualization, Data Curation, Visualization, Investigation Writing, Original draft preparation. **Faisal Abdi Osoble Osman:** Conceptualization, Data Curation. **Abdinasir Mohamed Elmi:** Data curation and reviewing. **Shuayb Moallim Ali Jama:** Supervision, Validation. **Yahye Garad Mohamed:** Data curation and reviewing. **Ismail Gedi Ibrahim:** Supervision, Validation. **Ahmed Adam Osman:** Supervision, Validation. **Mohamed Osman Dahir:** Conceptualization, Data Curation. **Abdirahman Mohamud Haji Ali:** Conceptualization, Data Curation. **Ismail Mohamud Abdullahi:** Conceptualization, Data Curation. **Nuh Mohamed yahye:** Data curation and reviewing. **Mohamed Salad kadiye:** Data curation and reviewing.

## Availability of data and materials

The data that support the findings of this study are available in Mogadishu Somali Turkey, Recep Tayyip Erdogan Training and Research Hospital information system. Data are however allowed to the authors upon reasonable request and with permission of the education and research committee.

## References

[1] Lancella A, Abbate G, Foscolo AM, Dosdegani R. (2008). Two unusual presentations of cervicofacial actinomycosis and review of the literature. Acta Otorhinolaryngol Ital.

[2] McCann A, Alvi SA, Newman J, Kakarala K, Staecker H, Chiu A (2019). Atypical form of cervicofacial actinomycosis involving the skull base and temporal bone. Ann Otol Rhinol Laryngol.

[3] Park JK, Lee HK, Ha HK, Choi HY, Choi CG. (2003). Cervicofacial actinomycosis: CT and MR imaging findings in seven patients. American Journal of Neuroradiology.

[4] Mishra A, Prabhuraj AR, Bhat D, Nandeesh BN, Mhatre R. (2019). Intracranial actinomycosis manifesting as a parenchymal mass lesion: A case report and review of literature. World Neurosurg.

[5] Chopra S, Christie M, Felimban H. (1995). Intracranial actinomycosis mimicking meningioma. Ann Saudi Med.

[6] Chatterjee RP, Shah N, Kundu S, Mahmud SA, Bhandari S. (2015). Cervicofacial actinomycosis mimicking osseous neoplasm: a rare case. J Clin Diagn Res.

[7] Swain SK. (2022). Actinomycosis in head and neck region: a review. Indian J Otolaryngol Head Neck Surg.

[8] Ansari F, Budania A, Rao M, Yadav T. (2022). Extensive actinomycosis with intracranial and mediastinal involvement: a therapeutic challenge. BMJ Case Rep.

[9] Ziemliński J, Boniukiewicz A, Braun M, Zagacki D, Kaczmarczyk D. (2022). Actinomycosis of the right maxillary sinus mimicking adenoid cystic carcinoma recurrence. Pol Przegląd Otorynolaryngol.

[10] O'Riordan D, Zermansky A, Bishop P, Taggart SCO. (2012). Actinomycosis masquerading as malignancy. Acute Med.

[11] Limaye H, Hinduja AA, Verma M, Oak P. (2021). A case of CNS actinomycosis: rarer than rare!. Neurol India.

